# Perceived needs and priorities of older persons in humanitarian crises: A scoping review of literature

**DOI:** 10.1007/s40520-025-03226-x

**Published:** 2025-11-17

**Authors:** Marco Canevelli, Yuka Sumi, Anshu Banerjee, Swagata Chetia, Arjan Gjonca, Hyobum Jang, Leila Khalid, Janus Maclang, Ignacio Salas, Ritu Sadana, Nicola Vanacore, Matteo Cesari

**Affiliations:** 1 Department of Human Neuroscience, Sapienza University, Rome, Italy; 2 National Centre for Disease Prevention and Health Promotion, Italian National Institute of Health, Rome, Italy; 3 Ageing and Health Unit, Department of Maternal, Newborn, Child, and Adolescent Health and Ageing, World Health Organization, Geneva, Switzerland; 4 Department of International Development, London School of Economics and Political Science, London, UK

**Keywords:** Aging, Geriatrics, Emergency, Disaster, Health

## Abstract

Humanitarian emergencies, including wars, forced displacements, sudden onset disasters, and pandemics, disproportionately affect older persons. This scoping review aims to map and synthesise existing studies on the care needs of older persons in such contexts, emphasising needs directly reported by the older persons themselves. A literature search was conducted on PubMed and EMBASE, focusing on qualitative studies that reported care needs of older persons in the context of humanitarian crises. The initial search yielded 4,409 articles, which were reduced to a final list of 27 articles after screening titles and abstracts, as well as an in-depth evaluation of full texts. The needs and priorities retrieved from the articles were clustered, taking into account the preparedness, response, and recovery phases of humanitarian crises to which they referred. Overall, findings indicated a broad spectrum of priorities reported by older persons. In particular, it was highlighted that the management of chronic conditions and functional impairments drove care needs. Furthermore, environmental barriers (including limited access to tailored information and inadequate transportation) and social issues (e.g., the need for support from others, disconnection from family, community, and cultural ties, and financial problems) are significant hurdles for older adults during crises. Unfortunately, qualitative studies reporting the perspectives of older people in the context of humanitarian crises still present substantial limitations, for which further research is required. Gathering evidence on the needs of older persons in humanitarian crises is essential. The scarcity of reliable data often results in their needs being overlooked during emergencies, leading to inadequate support.

**Supplementary Information:**

The online version contains supplementary material available at 10.1007/s40520-025-03226-x.

## Introduction

Population ageing is one of the major demographic phenomena observed globally, and is occurring at a particularly rapid pace in low- and middle-income countries (LMICs) [1]. However, the increase in life expectancy is not always matched by good health. The age-related accumulation of health deficits accentuates the individual’s vulnerability to stressors and increases their risk of experiencing adverse events [2].

In response to this background, the *United Nations Decade of Healthy Ageing (2021–2030)* seeks to transform the world into a better place to grow older, specifically by acting to fight ageism, creating age-friendly environments, and ensuring that older persons have access to a continuum of integrated care [3]. The older person (i.e., an individual who is over 60 years of age) is at the very centre of this global initiative. The active engagement of older people, represented in one of the four enablers outlined in the Decade’s Plan of Action [4], is essential to ensure that their experiences, preferences, and priorities are properly reflected and addressed in strategies and policy development [5]. Unfortunately, the voices of older persons often remain unheard, contributing to inadequacy and ineffectiveness in public health solutions. This is particularly evident when the older persons’ vulnerability is enhanced, such as in humanitarian crises [6].

Diverse humanitarian emergencies, including conflicts, forced displacements, sudden onset disasters, and pandemics, are occurring in many regions globally. In 2023, over 399 catastrophic events were recorded, leading to over 86,000 deaths and affecting 93 million people worldwide [7]. In the same year, over 117 million people were forcibly displaced [8]. Due to population ageing, these crises are increasingly involving older people. For example, the number of refugees aged 60 years and older has shown a tenfold increase in the last two decades [8]. Moreover, as recently learned from the COVID-19 outbreak, these events tend to have a disproportionate impact on older persons [9].

The health needs of older people in the context of humanitarian crises have been examined in two systematic reviews [10, 11]. Both studies indicated access to care, management of chronic conditions, and services for functional impairments as critical needs for older persons in humanitarian contexts. However, it is our view that this evidence only partially represents the older persons’ perspective. These research efforts were restricted to explicitly focus on LMICs. Moreover, the needs and priorities were not openly reported by older persons; instead, data collection in most of the retained studies adopted questionnaires using predefined selections of possible answers, potentially based on the investigator’s judgement rather than the older person’s viewpoint.

This scoping review aims to map and synthesise published literature on the care needs of older persons in humanitarian contexts. In particular, the study prioritises the identification of care needs based on what older persons actively and directly reported. By giving visibility to their perspective, it will be possible to improve the definition of what matters most to older persons facing such arduous scenarios and more accurately inform and adapt future research and humanitarian aid. Indeed, listening to their voices, perspectives, and experiences is pivotal to actively include older persons’ views in strategies addressing humanitarian crises [10].

## Methods

### Study design

A scoping review method was chosen to map the existing evidence and knowledge gaps on the needs and priorities of older persons in humanitarian crises. This approach was motivated by the relative novelty of the topic as well as the wide variety of humanitarian crises and potential care needs of older people. In particular, the self-reporting of older people’s needs and priorities in humanitarian settings has not yet been systematically explored.

The scoping review was conducted according to the Joanna Briggs Institute methodology [12], which is primarily used to generate evidence in healthcare-based research. It was reported following the Preferred Reporting Items for Systematic Reviews and Meta-Analyses extension for scoping reviews checklist [13].

## Research question

The present scoping review addressed the following research question: *What has been published in the scientific literature about the needs and priorities that older persons perceive as critical to improving care in the context of humanitarian crises?*

## Search strategy

A review of scientific literature was conducted by searching PubMed and EMBASE from inception to October 30, 2024. These databases were selected because they contain a comprehensive body of evidence that encompasses both the social and medical aspects of care. A detailed search strategy was developed by focusing on the central concepts of the research question, namely (1) needs and priorities of care, (2) older persons, and (3) humanitarian crises. A secondary search strategy was developed to incorporate papers that provided the caregivers’ perspective on the needs and priorities of the assisted older persons. The Boolean operator “OR” was used to combine multiple search terms. The three concepts were then combined with the Boolean operator “AND”. The search strategy was inspired by a previous systematic appraisal on humanitarian crises [14] and is summarized in the Online Resources, where the adopted search strings are also reported. The final search results were exported into Covidence, a web-based collaboration software platform that streamlines the production of systematic and other literature reviews [15]. Grey literature was not included in the review due to varying quality and reporting standards, potential for selection and publication bias, and inaccurate or incomplete reporting.

## Article selection

The following criteria were adopted to identify the articles of interest:

1. Articles had to directly report the needs and priorities as perceived by older persons (i.e., people aged 60 and over) or by carers of older persons who were unable to participate or express their needs (e.g., due to language barriers, sensory impairment, and cognitive deficits). For this purpose, qualitative studies were primarily targeted.
2. Articles had to focus on any type of humanitarian crisis, including conflicts, genocides, sudden onset disasters, epidemics, famines, displacements, or forced migrations.
3. Articles could report on older persons’ care needs and priorities encompassing both social and medical aspects.
4. Only articles published in English were considered.
5. No limitation was applied regarding publication date, geographical focus, or methodology.

A group of reviewers individually evaluated the relevance and pertinence of the publications retrieved from the search engines, considering their titles and abstracts. Subsequently, pairs of reviewers independently assessed the full texts of all potentially eligible studies. Any discrepancies in the article selection were discussed and resolved by consensus or with the involvement of a third reviewer.

## Data extraction and charting

A data-charting form was created to extract and collect the variables of interest. This included the digital object identifier (DOI), the type of study design, and the number of participants. The type of humanitarian crisis (e.g., pandemic, earthquake…), and when and where it happened were also recorded. Moreover, every sentence reporting a health need or care priority identified in the articles was included in the database, specifying whether it referred to the preparedness, response, and/or recovery phase of the emergency management.

### Clustering of needs and priorities

The sentences describing needs and priorities extracted from each paper were first listed horizontally in the data-charting form to facilitate their collection. Then, these raw entries were verticalised to support the standardisation of their wording. The resulting priorities were then represented in WiseMapping, an online mind map development software (https://www.wisemapping.com/). Three mind maps were produced to illustrate the care needs and priorities of older persons for each of the three phases of the humanitarian crisis.

## Results

**Fig. 1:**
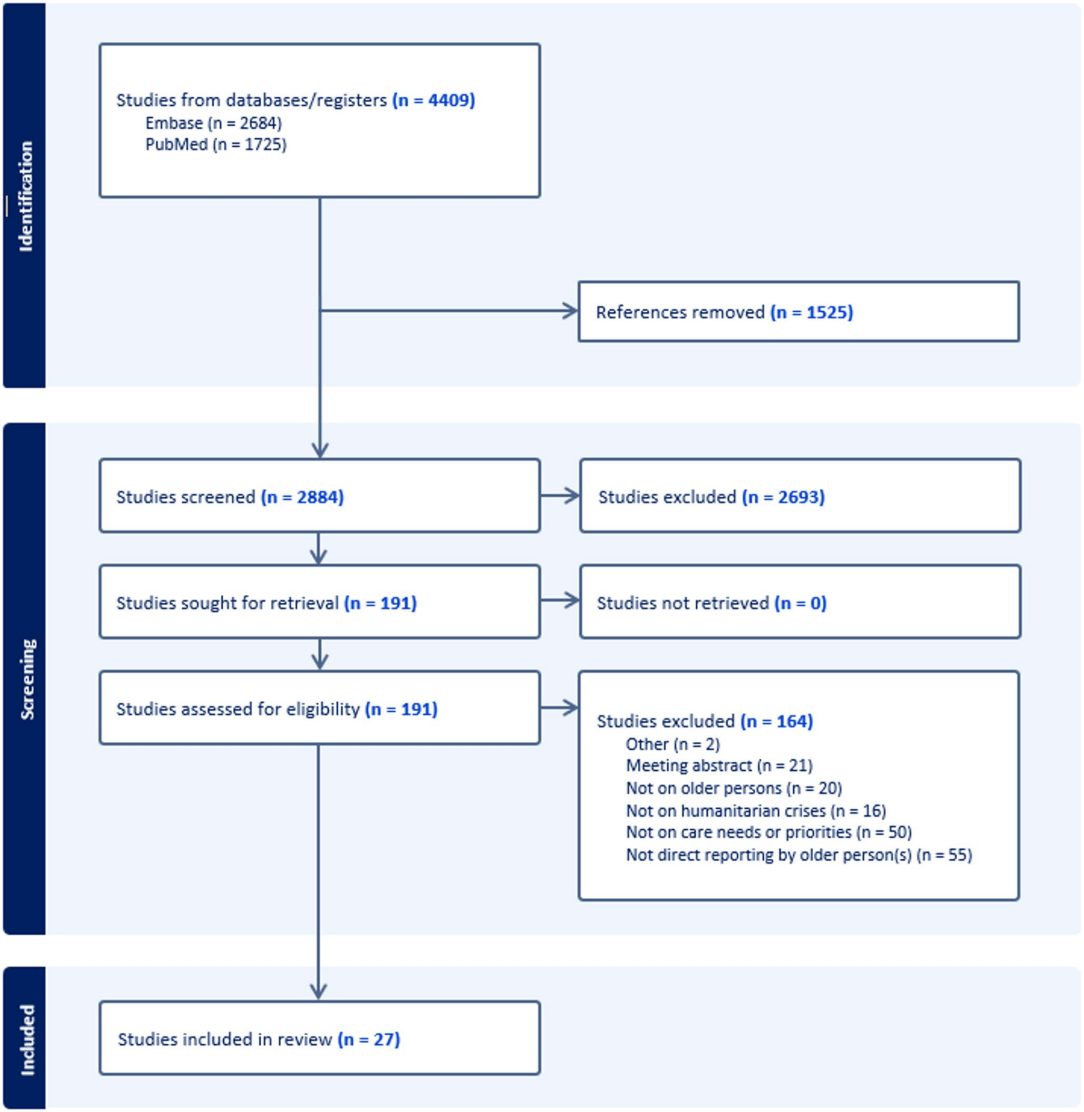
Flowchart describing the selection of articles of interest

**Fig. 2:**
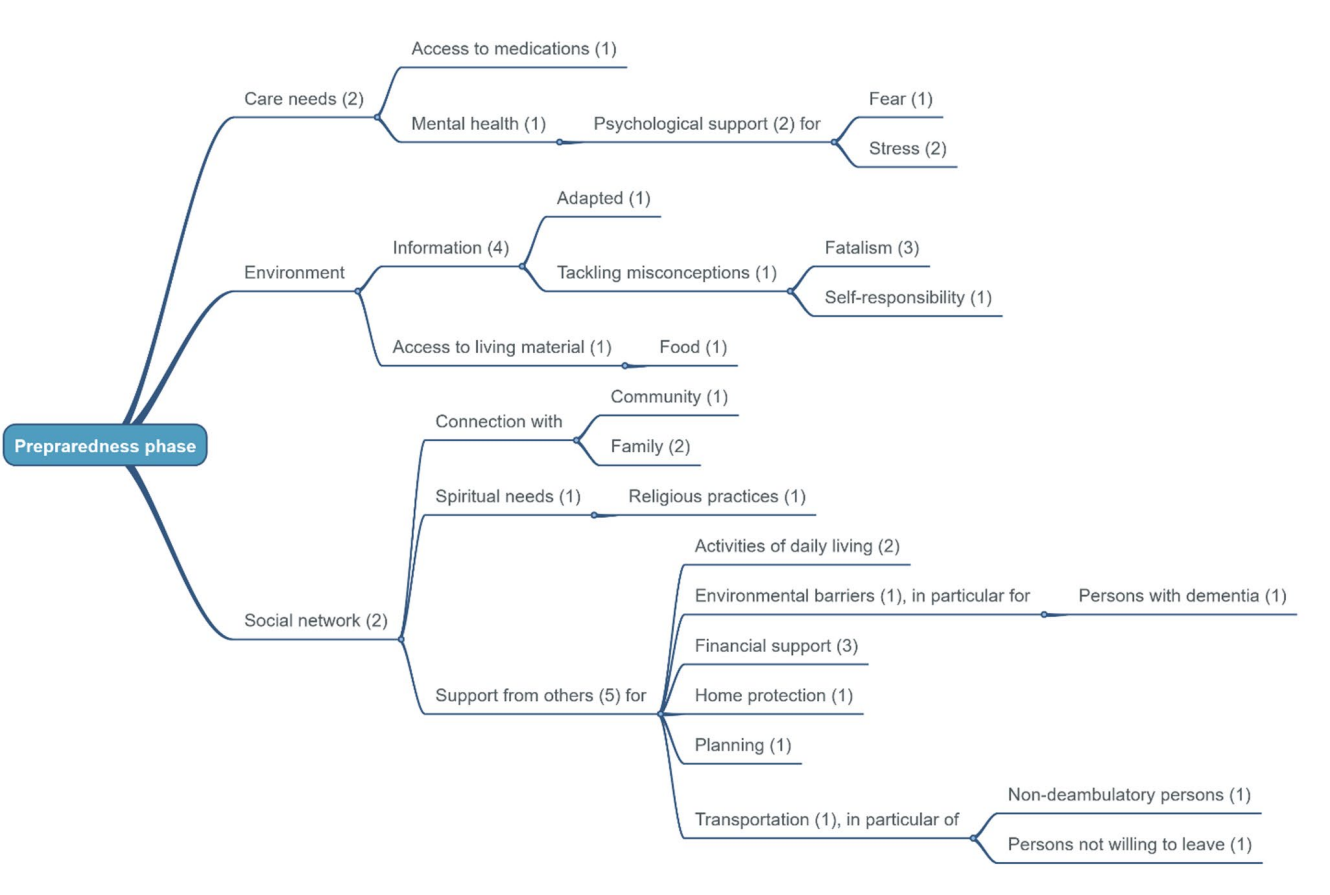
Results of clustering for the preparedness phase

**Fig. 3:**
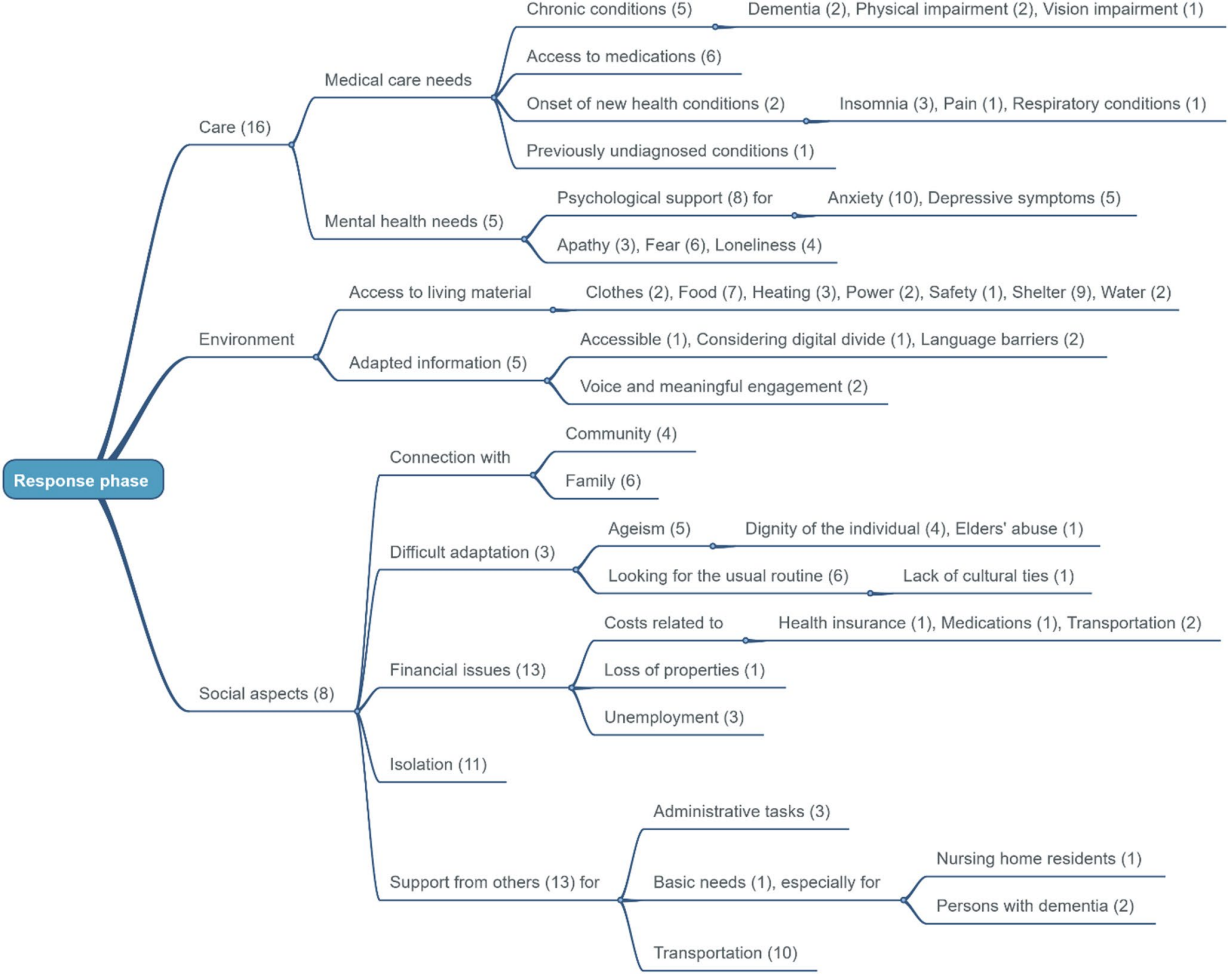
Results of clustering for the response phase

**Fig. 4:**
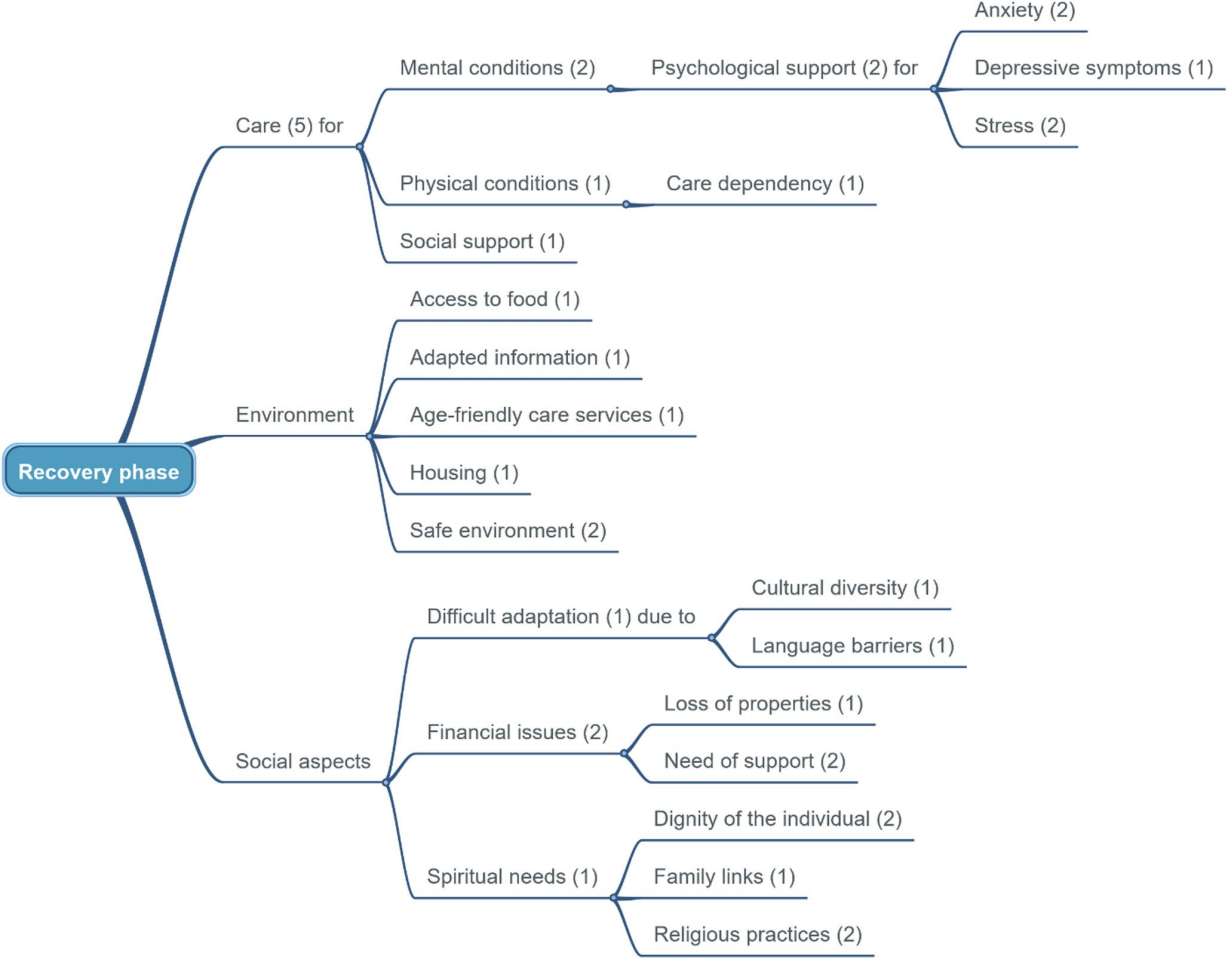
Results of clustering for the recovery phase

The initial search resulted in 4,409 articles, from which 1,525 duplicates were removed. A total of 2,884 articles underwent title and abstract screening, from which 2,693 were excluded. The remaining 191 articles underwent full review, with 164 excluded because they did not fulfil the predefined eligibility criteria. A final list of 27 articles was included in the final evidence synthesis (Fig. 1) [16–42]. The characteristics of these studies are summarised in Table 1. A total of 18 countries were represented in the studies, which explored eight humanitarian contexts: forced displacement (*n* = 8), pandemics (*n* = 7), earthquakes (*n* = 4), hurricanes (*n* = 4), tsunamis (*n* = 2), bushfires (*n* = 1), and floods (*n* = 1). Three articles focused on the preparation for unspecified disasters. Seventeen studies used qualitative methods, while ten utilised mixed methods. Five articles provided insights on the preparedness phases [21, 25, 30, 33, 41], 19 focused on the response phase [16–24, 26–29, 31, 32, 37, 39, 40, 42], and five on the recovery phase [16, 34–36, 38]. In total, 308 priorities were identified, with over two-thirds focusing on the response phase (*n* = 217), followed by 45 and 43 referring to the preparedness and recovery phases, respectively. Figures 2 and 3, and 4 show the clustered needs and priorities of older persons for each of the three phases.

**Table 1:** Summary characteristics of the retained studies*

**Countries**	Australia (*n* = 2) Belgium (*n* = 1) China (*n* = 1) Denmark (*n* = 1) Georgia (*n* = 1) Indonesia (*n* = 1)	Iran (*n* = 2) Ireland (*n* = 1) Lebanon (*n* = 2) New Zealand (*n* = 1) Nigeria (*n* = 1) Pakistan (*n* = 1)	Iran (*n* = 2) Ireland (*n* = 1) Lebanon (*n* = 2) New Zealand (*n* = 1) Nigeria (*n* = 1) Pakistan (*n* = 1)	Sri Lanka (*n* = 1) Sudan (*n* = 1) Sweden (*n* = 1) Syria (*n* = 2) Thailand (*n* = 1) USA (*n* = 9)
**Humanitarian Context**	Bushfires (*n* = 1) Disaster preparation (*n* = 3) Earthquake (*n* = 4) Flooding (*n* = 1)		Forced displacement (*n* = 8) Hurricane (*n* = 4) Pandemic (*n* = 7) Tsunami (*n* = 2)	
**Phases**	Preparedness (*n* = 5) Response (*n* = 19) Recovery (*n* = 5)			
**Method**	Qualitative (*n* = 17)		Mixed Methods (*n* = 10)	
**Participants**	Total, *n* = 2,983 Median (25th −75th percentile), *n* = 50 (20–155)			
**Priorities**	Total, *n* = 308 Preparedness (*n* = 45) Response (*n* = 217) Recovery (*n* = 43)			

* The numbers in the table may not add up to the total of 27 retained articles, as the same study could provide results referring to different countries, contexts and phases

## Preparedness phase

The primary cluster of priorities reported by older persons centres on the need for support from others [25, 30, 33, 41]. Participants frequently indicated that they require assistance with activities of daily living, financial support, and help with transportation [25, 30, 33, 41]. Another significant cluster of needs highlighted in participants’ responses relates to the provision of meaningful information to adequately prepare [21, 25, 30, 41]. There was a clear call for more comprehensive and tailored communication methods that consider the potential limitations of older persons and common misconceptions (e.g., the idea that individuals are solely responsible for their well-being). Additionally, three articles noted the fatalism often present in older adults [21, 25, 41].

Access to medications was also indicated as a care need to consider [25]. Furthermore, stress and fear were of particular concern for older persons in preparation for a crisis, prompting to the request for psychological support [25, 30, 33].

Older people also mentioned the importance of social networks, emphasising their need to remain connected with family and community [25, 33]. Additionally, they identified spiritual needs and religious practices as significant aspects to consider in preparation for a catastrophic event [33].

### Response phase

Older people repeatedly indicated health care needs during humanitarian crises. They reported having limited access to necessary medications [16, 19, 20, 22, 24, 42] and that the difficulties associated with crisis frequently worsen their chronic conditions (e.g., dementia, physical impairment, vision impairment) [16, 21, 26, 37, 40, 42]. Additionally, new health issues, such as insomnia [18, 19, 29] and pain [26], can arise. Mental health conditions, including depression, anxiety, fear, and loneliness, determine the requests for psychological support [18–20, 23, 24, 27–29, 31, 32, 37, 42].

Many depend on others for support, including assistance with bureaucratic obstacles that hinder their access to aid from humanitarian agencies [17, 18, 24, 26, 28, 32, 39, 42]. In this context, transportation emerged as another significant hurdle due to both the person’s physical impairments and environmental barriers (e.g., cost, road blockages) [16–19, 21, 26, 28, 31, 39, 42].

Participants also described financial threats due to the loss of employment and properties, leading to the unaffordability of health insurance and medications [17, 19, 21–23, 26, 29, 31, 37, 39]. Older persons reported considerable hardships securing basic living resources, such as clothing, food, heating, and safety [17–20, 26, 31, 37]. In particular, difficulties related to accessing and maintaining shelters were frequently reported [17, 19, 21, 23, 26, 29, 31, 39].

Older people perceived disruptions to daily routines as particularly burdensome [18, 24, 29, 40, 42]. They mentioned difficulties in coping with the new scenario and sought emotional support and ways to maintain social connections. Finally, some older persons complained about experiencing age-based discrimination and the underprioritization of their needs during the humanitarian crisis [19, 22, 26–28].

### Recovery phase

Humanitarian crises directly affect older persons’ physical health by exacerbating chronic illnesses and disrupting access to healthcare, even in the long term [16, 34, 38]. Their well-being is linked to the capacity to remain engaged in daily activities, such as walking and socialising. Psychosocial support addressing anxiety, depression, loneliness, and stress is indicated by older persons as critical in the aftermath of a catastrophic event [35, 36, 38].

Older people also reported facing financial concerns, housing difficulties, and food insecurity during the recovery phase [34, 35, 38]. Transportation challenges and economic problems can further hinder their access to nutritious food. Older persons also indicated spiritual well-being as a key aspect to prioritise comfort and distraction during and following uncertain times [34, 38].

Finally, the results of the three mindmaps were consolidated into a single output, organised into three domains: (1) Care, (2) Environments, and (3) Social aspects. Table 2 accordingly describes the primary needs and priorities reported by older persons in five or more of the retrieved articles.

**Table 2:** Overview of the needs and priorities most frequently reported* by older persons in the context of humanitarian crises

Domain	Primary needs and priorities
Care	- Management of chronic conditions - Access to medications - Need for psychological support
Environments	- Access to food - Age-friendly shelters - Adapted communication
Social aspects	- Connect with the community and the family - Financial support - Promote adaptation by: o Combatting ageism o Reducing isolation o Facilitating a return to the usual routine - Support from others for o Transportation o Basic needs

* The needs and priorities included in the table were identified from those reporting five or more citations from the analysed literature after consolidation of the results presented in Figs. 2, 3 and 4

## Discussion

To our knowledge, this study represents the first systematic effort to describe the care needs and priorities as perceived and reported by older persons in the context of humanitarian crises. This important topic has received too little attention, especially if we consider the ongoing demographic changes and the growing burden of conflicts, forced displacement, sudden onset disasters, and pandemics.

As mentioned, previous attempts to capture older persons’ needs in humanitarian crises were focused on LMICs and primarily relied on input from quantitative research (i.e., predefined answers) proposed to participants [10, 11, 43]. Not surprisingly, the results were particularly focused on care delivery for chronic conditions and functional limitations. This type of need is also evident in our results, where physical and mental conditions, as well as the management of diseases, were repeatedly reported (*“Difficulty accessing medical care”*, *“Treatment plans for cancer postponed indefinitely by fires then COVID”*, or *“Could not proceed with spinal or ankle surgery”* [29]). A recent scoping review, considering both quantitative and qualitative studies on the vulnerabilities and needs of older populations affected by humanitarian crises, identified the most relevant body of literature on mental health disorders [43]. Nevertheless, although relevant, these issues do not entirely reflect what older persons may prioritise and value most. Based on more qualitative research, our findings indicate that aspects such as access to information, spiritual life, sociocultural ties, transportation, financial security, and involvement in decision-making are critical for older persons facing humanitarian crises.

A few examples extrapolated from the retained studies can illustrate the broadness of older people’s priorities in these contexts. Focus groups conducted in communities of Congolese refugees in the United States highlighted the risk of elder abuse while describing communication and transportation as major issues, also for accessing care (*“English [language]*,* transportation*,* not knowing how to drive. Hospital ride and no interpreters is my major problem”* [39]). The complexity of communicating with older persons, especially during emergencies, can be represented by the paradoxical case reported in a study examining the reactions of older persons during Hurricane Sandy in the United States. Two older persons were surprised by the flooding of their house and were unaware they had to leave their home, even though they knew it was in a mandatory evacuation zone. One of the two explained: *“Because no one knocked on our door*,* and I was home all day!”* [30]. Communication difficulties (also due to the digital divide and financial issues related to phone/internet connection) and the need for support from others (especially family members) can be perceived in the words of older persons who were internally displaced in North Nigeria: *“…I have to eat first before I talk about buying a phone and recharging it with airtime. Children help us”* [22]. A 70-year-old individual, who experienced the Bam earthquake in Iran in 2003, explained coping through the challenging situation with the support of religion (“*I read the Quran more than before*,* and it is calming*”) [34]. In a study of Vietnamese refugees, one participant expressed the importance of cultural ties with the home country and perpetuating traditions to following generations, saying: “*I tell them [grandchildren] stories of Vietnam; I tell them how it was when I was their age. I remind them of Vietnam. I cannot possibly forget it. Because we had to leave*,* we do not forget our culture or lose it*” [38]. An older Syrian refugee in Lebanon described his challenges in fixing his shelter: *“It was raining and the caravan was leaking. I climbed up to put something to stop the leakage*,* and it was windy*,* […] I fell from the caravan to the ground. On the cement. […]. Afterwards*,* I found myself failing to get up. I deteriorated until I found myself in a wheelchair”* [17]. Another study conducted in a group of internally displaced older Georgian persons noted their feelings of isolation and abandonment, leading to apathy (*“They are inert and emotionless. As if they do not have a desire to live anymore”*) and feeling to be *“a burden to family”* [37].

At the same time, it is noteworthy that the fatalism presented by some older persons facing a humanitarian crisis is an aspect to consider as potentially affecting the effectiveness of interventions. For example, in a study focused on preparedness strategies against hurricanes in the United States, a participant said, *“I’m prepared for everything*,* really. I’ve been prepared all my life. How much time do I have left? You know it’s hard for a hurricane being the biggest catastrophe. Anybody that survived two wars and the Great Depression can pretty much take care of themselves”* [41].

Humanitarian principles dictate that assistance and protection be provided based on need and without any form of discrimination. However, older persons risk being overlooked in receiving humanitarian assistance as their needs are not seen and understood. Some of the difficulties they face when preparing for, responding to, or recovering from humanitarian crises (e.g., loneliness, financial concerns, transportation issues, lack of basic living resources, insomnia) are not inherently age-related and may also affect younger individuals. However, the increased vulnerability that often characterises older persons due to the high prevalence of health and social conditions may particularly enhance and emphasise the relevance of these needs and priorities for this specific population. Collecting evidence and engaging with older people is essential for identifying their priorities and promoting access to responsive humanitarian strategies. This engagement is crucial for developing and implementing the *Humanitarian Inclusion Standards for Older People and People with Disabilities*, coordinated by the Age and Disability Capacity Programme [44], a guidance aimed at giving more visibility to those needs that are often overlooked during a crisis. From this perspective, our findings may help tailor interventions in humanitarian crises to more effectively respond to the older persons’ priorities. Such an approach calls for cross-sectoral collaboration and a strong commitment from multiple actors, encompassing humanitarian actors, governmental and non-governmental organisations, experts, and older persons themselves. By building strong partnerships among these stakeholders, we can develop comprehensive strategies prioritising the inclusion and support of older persons in challenging contexts, ultimately leading to more effective and equitable responses. In this context, the overview derived from our consolidated findings (Table 2) may provide insights into improving the approach to and assessment of older persons in humanitarian settings by streamlining and unifying their needs and priorities. Conducting more interpretive research, such as phenomenological studies and in-depth interviews, can help deepen our understanding of older people’s needs. For example, statements from older individuals that were sometimes interpreted as fatalism might instead reflect pragmatism shaped by a lifetime of overcoming adversity.

This review has several limitations. First, it relies solely on literature published in English. Additionally, we focused exclusively on scientific literature, leaving out grey literature. Whereas these methodological choices may have resulted in missing valuable insights, they also contributed to the robustness of the information gathered. Our objective to prioritise studies describing the needs openly and directly presented by older persons proved to be difficult to achieve. This was due to the heterogeneous reporting of the methodologies and results in the retrieved evidence. For example, it was not always possible to establish how much the needs were burdening the individual and/or prioritised by the investigator. This may have potentially influenced the accurate selection of articles or needs. However, we believe that our results -emerging from a scoping review methodology- fulfil the objective of our work, which was to comprehensively explore the available evidence to provide a picture of the older persons’ diverse needs and priorities in the face of humanitarian crises. Our analyses allowed us to identify and present the needs and priorities of older persons during three different phases of humanitarian crises. However, the available data are still relatively scarce and fragmented, which limits our ability to conduct more detailed secondary analyses. For instance, it would have been interesting to stratify our findings based on the characteristics of participants, the type of event that caused the humanitarian crisis, the countries affected, and the socioeconomic context (e.g., income levels). Older persons experiencing humanitarian crises can differ widely in health, economic status, sociocultural characteristics, and previous exposure to similar experiences. Overlooking such heterogeneity could lead to the mistaken assumption that all older people are similarly vulnerable during crises. Additionally, the external factors (e.g., the country’s inherent resilience and availability of resources) can significantly shape the impact of the crisis on the older population and must be adequately considered. It might be argued that our findings are based on studies from a limited number of countries (*n* = 18), frequently characterised by high income. This aspect will affect the direct, global generalisability of our results. The bias of scientific literature towards high-income countries and the underrepresentation of older persons in research activities, even in normal times, **is** widely documented [45, 46]. In this context, our attempt to give voice and visibility to the most vulnerable population in severely adverse situations also aimed at soliciting more inclusive research.

In conclusion, gathering comprehensive and robust evidence on the specific needs and priorities of older persons, particularly those most vulnerable, such as those affected by humanitarian crises, is crucial. The current scarcity of reliable data often leads to this vulnerable group being overlooked and marginalised during emergencies, resulting in inadequate support. To address these shortcomings, it is essential to actively involve older persons in the planning and implementation of humanitarian responses. Their insights and experiences can greatly enhance the effectiveness and inclusivity of these efforts.

## Supplementary Information

Below is the link to the electronic supplementary material.

Supplementary Material 1

## Data Availability

No datasets were generated or analysed during the current study.
