# Supplementary material for: Perceived needs and priorities of older persons in humanitarian crises: A scoping review of literature
Source: Aging Clin Exp Res. 2025 Nov 17;37(1):328. doi: 10.1007/s40520-025-03226-x (PMC12628393; doi:10.1007/s40520-025-03226-x)
Supplement: Supplementary file 1 — Supplementary Material 1 [file 40520_2025_3226_MOESM1_ESM.pdf]

Perceived need and priorities of older persons in humanitarian crises: A scoping review of literature

Supplementary Material

Search strategy key terms

	AND		
**OR**	Care<br>Need*<br>Priorit*	Older person*<br>Older people<br>Elder*<br>Older subject*<br>Older individual*<br>Older population*<br>Aging population*<br>Ageing population*<br>***WITH AND WITHOUT***<br>*Caregiver**<br>*Care giver**<br>*Carer**	Humanitarian<br>Disaster*

Search strategies

PubMed (without caregivers)

(("Humanitarian*"[tiab] OR "Disaster*"[tiab] OR "Crisis"[tiab] OR "Mass Casualty Incident" [tiab] OR "Mass fatality"[tiab] OR "War"[tiab] OR "Armed conflict"[tiab] OR "Conflict"[tiab] OR "Genocide"[tiab] OR "Ethnic cleansing"[tiab] OR "Natural disaster"[tiab] OR "Epidemic" [tiab] OR "Disease outbreak"[tiab] OR "Mudslide"[tiab] OR "Earthquake"[tiab] OR "Flood*" [tiab] OR "Landslide"[tiab] OR "Volcano"[tiab] OR "Tidal wave"[tiab] OR "Tidalwave"[tiab] OR "Tsunami"[tiab] OR "Cyclonic Storm"[tiab] OR "Typhoon"[tiab] OR "Hurricane"[tiab] OR "Cyclone"[tiab] OR "Drought"[tiab] OR "Starvation"[tiab] OR "Famine"[tiab] OR "Relief Work*"[tiab] OR "Aid work*"[tiab] OR "Disaster medicine"[tiab] OR "Conflict Affected*"[tiab] OR "Conflict-Affected*"[tiab] OR "Forced Migrant*"[tiab] OR "Forced Migration"[tiab] OR "Forcibly displaced"[tiab] OR "Refugee*"[tiab] OR "Asylum"[tiab] OR "Asylum seeker*"[tiab] OR "Internal* displace*"[tiab] OR "IDP"[tiab] OR "Displaced person*"[tiab] OR "Displaced population*"[tiab]) AND ("older person*"[tiab] OR "older people"[tiab] OR "elder*"[tiab] OR "older individual*"[tiab] OR "older subject*"[tiab] OR "aging population*"[tiab] OR "ageing population*"[tiab] OR "healthy ageing"[tiab] OR "healthy aging"[tiab]) AND ("need*"[tiab] OR "priorit*"[tiab] OR "value*"[tiab] OR "feeling*"[tiab] OR "perception*" [tiab])) AND ((humans[Filter]) AND (english[Filter]))

PubMed (with caregivers)

(("Humanitarian*"[tiab] OR "Disaster*"[tiab] OR "Crisis"[tiab] OR "Mass Casualty Incident" [tiab] OR "Mass fatality"[tiab] OR "War"[tiab] OR "Armed conflict"[tiab] OR "Conflict"[tiab] OR "Genocide"[tiab] OR "Ethnic cleansing"[tiab] OR "Natural disaster"[tiab] OR "Epidemic" [tiab] OR "Disease outbreak"[tiab] OR "Mudslide"[tiab] OR "Earthquake"[tiab] OR "Flood*" [tiab] OR "Landslide"[tiab] OR "Volcano"[tiab] OR "Tidal wave"[tiab] OR "Tidalwave"[tiab] OR "Tsunami"[tiab] OR "Cyclonic Storm"[tiab] OR "Typhoon"[tiab] OR "Hurricane"[tiab] OR "Cyclone"[tiab] OR "Drought"[tiab] OR "Starvation"[tiab] OR "Famine"[tiab] OR "Relief Work*"[tiab] OR "Aid work*"[tiab] OR "Disaster medicine"[tiab] OR "Conflict Affected*"[tiab] OR "Conflict-Affected*"[tiab] OR "Forced Migrant*"[tiab] OR "Forced Migration"[tiab] OR "Forcibly displaced"[tiab] OR "Refugee*"[tiab] OR "Asylum"[tiab] OR "Asylum seeker*"[tiab] OR "Internal* displace*"[tiab] OR "IDP"[tiab] OR "Displaced person*"[tiab] OR "Displaced population*"[tiab])

AND ((("older person*"[tiab] OR "older people"[tiab] OR "elder*"[tiab] OR "older individual*"[tiab] OR "older subject*"[tiab] OR "aging population*"[tiab] OR "ageing population*"[tiab] OR "healthy ageing"[tiab] OR "healthy aging"[tiab])) OR (("older person*"[tiab] OR "older people"[tiab] OR "elder*"[tiab] OR "older individual*"[tiab] OR "older subject*"[tiab] OR "aging population*"[tiab] OR "ageing population*"[tiab] OR "healthy ageing"[tiab] OR "healthy aging"[tiab]) AND (“caregiver*”[tiab] OR “care giver*” [tiab] OR “carer*”[tiab]))) AND ("need*"[tiab] OR "priorit*"[tiab] OR "value*"[tiab] OR "feeling*"[tiab] OR "perception*" [tiab])) AND ((humans[Filter]) AND (english[Filter]))

EMBASE (without caregivers)

(('humanitarian*':ab,ti OR 'disaster*':ab,ti OR 'crisis':ab,ti OR 'mass casualty incident':ab,ti OR 'mass fatality':ab,ti OR 'war':ab,ti OR 'armed conflict':ab,ti OR 'conflict':ab,ti OR 'genocide':ab,ti OR 'ethnic cleansing':ab,ti OR 'natural disaster':ab,ti OR 'epidemic':ab,ti OR 'disease outbreak':ab,ti OR 'mudslide':ab,ti OR 'earthquake':ab,ti OR 'flood*':ab,ti OR 'landslide':ab,ti OR 'volcano':ab,ti OR 'tidal wave':ab,ti OR 'tidalwave':ab,ti OR 'tsunami':ab,ti OR 'cyclonic storm':ab,ti OR 'typhoon':ab,ti OR 'hurricane':ab,ti OR 'cyclone':ab,ti OR 'drought':ab,ti OR 'starvation':ab,ti OR 'famine':ab,ti OR 'relief work*':ab,ti OR 'aid work*':ab,ti OR 'disaster medicine':ab,ti OR 'conflict affected*':ab,ti OR 'conflict-affected*':ab,ti OR 'forced migrant*':ab,ti OR 'forced migration':ab,ti OR 'forcibly displaced':ab,ti OR 'refugee*':ab,ti OR 'asylum':ab,ti OR 'asylum seeker*':ab,ti OR 'internal* displace*':ab,ti OR 'idp':ab,ti OR 'displaced person*':ab,ti OR 'displaced population*':ab,ti) AND ('older person*':ab,ti OR 'older people':ab,ti OR 'elder*':ab,ti OR 'older individual*':ab,ti OR 'older subject*':ab,ti OR 'aging population*':ab,ti OR 'ageing population*':ab,ti OR 'healthy ageing':ab,ti OR 'healthy aging':ab,ti) AND ('need*':ab,ti OR 'priorit*':ab,ti OR 'value*':ab,ti OR 'feeling*':ab,ti OR 'perception*':ab,ti) AND [humans]/lim AND [english]/lim

EMBASE (with caregivers)

(('humanitarian*':ab,ti OR 'disaster*':ab,ti OR 'crisis':ab,ti OR 'mass casualty incident':ab,ti OR 'mass fatality':ab,ti OR 'war':ab,ti OR 'armed conflict':ab,ti OR 'conflict':ab,ti OR 'genocide':ab,ti OR 'ethnic cleansing':ab,ti OR 'natural disaster':ab,ti OR 'epidemic':ab,ti OR 'disease outbreak':ab,ti OR 'mudslide':ab,ti OR 'earthquake':ab,ti OR 'flood*':ab,ti OR 'landslide':ab,ti OR 'volcano':ab,ti OR 'tidal wave':ab,ti OR

'tidalwave':ab,ti OR 'tsunami':ab,ti OR 'cyclonic storm':ab,ti OR 'typhoon':ab,ti OR 'hurricane':ab,ti OR 'cyclone':ab,ti OR 'drought':ab,ti OR 'starvation':ab,ti OR 'famine':ab,ti OR 'relief work*':ab,ti OR 'aid work*':ab,ti OR 'disaster medicine':ab,ti OR 'conflict affected*':ab,ti OR 'conflict-affected*':ab,ti OR 'forced migrant*':ab,ti OR 'forced migration':ab,ti OR 'forcibly displaced':ab,ti OR 'refugee*':ab,ti OR 'asylum':ab,ti OR 'asylum seeker*':ab,ti OR 'internal* displace*':ab,ti OR 'idp':ab,ti OR 'displaced person*':ab,ti OR 'displaced population*':ab,ti) AND ((('older person*':ab,ti OR 'older people':ab,ti OR 'elder*':ab,ti OR 'older individual*':ab,ti OR 'older subject*':ab,ti OR 'aging population*':ab,ti OR 'ageing population*':ab,ti OR 'healthy ageing':ab,ti OR 'healthy aging':ab,ti)) OR

(('older person*':ab,ti OR 'older people':ab,ti OR 'elder*':ab,ti OR 'older individual*':ab,ti OR 'older subject*':ab,ti OR 'aging population*':ab,ti OR 'ageing population*':ab,ti OR 'healthy ageing':ab,ti OR 'healthy aging':ab,ti)) AND (‘caregiver*’:ab,ti OR ‘care giver*’:ab,ti OR ‘carer*’:ab,ti))) AND

('need*':ab,ti OR 'priorit*':ab,ti OR 'value*':ab,ti OR 'feeling*':ab,ti OR 'perception*':ab,ti) AND

[humans]/lim AND [english]/lim

Table S1. Characteristics of the included studies

Reference	Setting	Country of the event	Event (year)	Participants	Phase of humanitarian crisis
Adams et al., 2011	Hurricane, flooding	USA	Hurricane Katrina (2005)	n = 163 interviews (58 of them 65+)	Response and Recovery
Al Omari et al., 2024	Forced displacement	Syria-Lebanon	Humanitarian crisis (2019)	n = 461 (50+); n = 14 interviews	Response
Aurtzki et al., 2024	Earthquake	Indonesia	Lombok earthquake (2018)	n = 16	Response
Chan et al., 2009	Earthquake	Pakistan	Kashmir earthquake (2006)	n = 125 questionnaires	Response
Chemali et al., 2018	Forced displacement	Syria-Lebanon	Humanitarian crisis (2015)	n = 66 (65+)	Response
Duggan et al., 2010	Tsunami	Sri Lanka-USA	Indian Ocean Tsunami (2004)	n = 17	Preparedness and Response
Ekoh et al., 2023	Forced displacement	Nigeria	Boko-Haram group insurgency (2009)	n = 14	Response
Forouzan et al., 2013	Earthquake	Iran	Earthquake (2006)	n = 6	Response
Frey et al., 2024	Pandemic	New Zealand	COVID-19 lockdown (2020)	n = 10 unpaid caregivers of older people living with dementia	Response
Gershon et al., 2017	Disaster preparation	USA	Not applicable	n = 50	Preparedness
Godfrey et al., 1989	Forced displacement	Sudan	Ethiopia war (1984-1985)	n = 383 refugees	Response
Gustavsson et al., 2024	Pandemic	Sweden	COVID-19 lockdown (2020)	n = 41	Response
Guzman et al., 2023	Pandemic	Ireland	COVID-19 lockdown (2021)	n = 57	Response
Halcomb et al., 2023	Pandemic and Bushfires	Australia	COVID-19 lockdown (2020) and bushfires (2019-2020)	n = 155	Response
Heagele et al., 2021	Disaster preparation	USA	Not applicable	n = 33	Preparedness
Heid et al., 2017	Hurricane	USA	Hurricane Sandy (2012)	n = 20	Response

Kaelen et al., 2021	Pandemic	Belgium	COVID-19 lockdown (2020)	n = 56 nursing home residents	Response
Kanokthet et al., 2018	Disaster preparation	Thailand	Not applicable	n = 657	Preparedness
Khodadadi et al., 2018	Earthquake	Iran	Earthquakes (2003 and 2005)	n = 12	Recovery
Nielsen et al., 2018	Forced displacement	Denmark	Not applicable	n = 21	Recovery
Pietrzak et al., 2012	Hurricane	USA	Hurricane Ike (2008)	n = 193	Recovery
Singh et al., 2018	Forced displacement	Georgia	South Ossetia conflict (2008)	n = 120	Response
Strumpf et al., 2001	Forced displacement	USA	Multiple events	n = 52	Recovery
Sudha et al., 2022	Forced displacement	USA	Multiple events	n = 30	Response
Thompson et al., 2024	Pandemic and Bushfires	Australia	COVID-19 pandemic and bushfires (2019-2020)	n = 19	Response
Wang et al., 2018	Hurricane	USA	Not applicable	n = 30	Preparedness
Wu et al., 2021	Pandemic	China	COVID-19 pandemic	n = 19	Response
